# Photobiomodulation by low-level laser therapy in patients with obstructive sleep apnea: Study protocol clinical trial (SPIRIT compliant)

**DOI:** 10.1097/MD.0000000000019547

**Published:** 2020-03-20

**Authors:** Fernanda Cristina Ferreira de Camargo, José Roberto DeMoura, Felipe Xerez Cepeda, Marilia de Almeida Correia, Reginaldo Ceolin Nascimento, Lucas Fortes-Queiroz, Fabiana Gonçalves Ferreira, Renata Kelly da Palma, Maria Fernanda Hussid, Maria Cristina Chavantes, Ivani Credidio Trombetta

**Affiliations:** a Graduate Program in Medicine, University Nove de Julho; b School of Physical Education, Military Police of São Paulo State, São Paulo, Brazil.

**Keywords:** biophotonics, index of apnea-hypopnea, laser therapy, metabolic syndrome, sleep-disordered breathing, snoring, sympathovagal balance

## Abstract

Obstructive sleep apnea (OSA) increases morbidity and mortality and it is associated with an increased cardiovascular risk. The gold standard treatment for OSA is positive airway pressure therapy (CPAP). However, it is an expensive treatment and several patients do not adapt to CPAP.

**Goal:**

The researchers will verify the effects of low-level laser therapy (LLLT) on OSA, when applied to the soft palate and on the tongue base.

**Methods:**

The researchers will select individuals of both sexes aged 30 to 60 years old who are sedentary and that present a high risk of OSA by the Berlin questionnaire. The evaluations pre and post interventions will be polysomnography; anthropometric and body composition measurements (Bioimpedance); metabolic syndrome risk factors (International Diabetes Federation); physical capacity (VO_2_ peak at the cardiopulmonary exercise test, CPET); endothelial function (flow-mediated dilatation, FMD); autonomic control (heart rate variability and sympathovagal balance). Those diagnosed with moderate and severe OSA (apnea/hypopnea index, AHI ≥15 events/h) will be invited to participate in the study and they will be randomized into 2 groups: LLLT treatment or placebo (C). The LLLT group will receive applications at 8 points on the soft palate and on the base of the tongue for 8 seconds for each point. The applications of LLLT will occur twice a week, with a minimum interval of 2 days between the applications for 2 months, when using a Therapy Plus NS 13678 Laser. The C group will have similar applications, but with the device turned off.

**Expected Results:**

In the individuals with OSA, photobiomodulation through LLLT will decrease the AHI. Additionally, when LLLT is applied in the oral cavity, a highly vascularized region, this may cause improvements in the vascular function and in the autonomic and hemodynamic control.

**Ethics and Dissemination:**

This protocol was approved by the Research Ethics Committee of the Nove de Julho University, São Paulo, Brazil, on the date of March 11, 2019 (CAAE: 06025618.2.0000.5511 - Acceptance Number: 3.191.077). This trial has been registered with the Brazilian Registry of Clinical Trials (REBEC TRIAL RBR-42v548). This study is not yet recruiting. Issue date: November 4, 2019.

## 1 Introduction

Biophotonics has many possibilities of acting in medicine. Previous studies in animal experimentation^[1]^ and in humans have shown that low-intensity laser therapy interferes positively with healing, inflammation, and oxidative stress,^[2]^ besides provoking analgesia.^[3]^ Additionally, recent studies have pointed to the possibility of high and medium intensity laser therapy in the treatment of patients with obstructive sleep apnea (OSA).^[4]^ This infers that a few applications of this therapy are sufficient to cause an increase in collagen in the soft palate of the patients, a region more susceptible to collapse during sleep. However, in the applications of high and medium intensity laser, the limit between provoking benefits and injuring the sensitive area that is constituted by the mucosa makes it difficult to investigate, especially if it is a future therapeutical application. In this sense, photobiomodulation with clinically applied LLLT may be a safe option for the treatment of snoring and OSA.

The gold standard for the diagnosis of OSA is polysomnography, whereas the treatment is positive airway pressure (CPAP) therapy.^[5]^ However, it is an expensive treatment and it is not covered by the public health service. In addition, several patients do not adapt to CPAP, or even to the intraoral device, while at the same time, they do not consistently adhere to the physical training. These patients, in particular, may benefit from a new therapy. In fact, although CPAP has proven its effectiveness, it is a palliative treatment. In this sense, LLLT, if it proves to be efficient, could be an adjuvant therapy to the conventional treatments with CPAP and the intra-oral device. LLLT may be a new therapeutical option that is safe, easy to apply, and inexpensive (when compared with CPAP and even intra-oral). In addition, it has been demonstrated in mice that LLLT in the vascularized region increases the parasympathetic activity, generating a deceleration of the heart rate, with a decrease in blood pressure, a decrease in sympathetic nervous activity, and a decrease in blood glucose. There is a strong association of OSA with snoring, obesity, and systemic arterial hypertension (SAH), predisposing these patients to a greater cardiovascular risk.

Thus, in this study, the researchers will test the hypothesis that LLLT, when it is applied to the soft palate, decreases the phallus collapsibility, snoring, and the apnea/hypopnea index. In addition, if it improves the autonomic and hemodynamic control in patients with moderate and severe OSA.

The study's anxiety is to check whether the LLLT parameters of this protocol are sufficient to benefit sleep apnea patients. This protocol was designed based on preliminary studies when using validated questionnaires, polysomnography, and spirometry.

In this study, data from other parameters related to sleep disorders will also be collected, which will be analyzed before and after the treatments, in order to allow for the Brazilian population to use a new approach for snoring and sleep apnea. In this way, the research will be aimed at evaluating and comparing the individuals who snore and who suffer from OSA, when submitted to low-intensity laser photobiomodulation, for 2 months over 24 sessions. This will be performed using polysomnography, modified Mallampati, spirometry, and when associated with the questionnaires of Berlin, the Epworth Sleepiness Scale, and the Stress Questionnaire. The groups will be compared, in order to analyze the possibilities of improvements in snoring, apnea, the hypopnea index, the autonomic and hemodynamic conditions, and the blood inflammatory markers when snoring and with OSA. This will be so as to enable the use of this low-intensity laser photobiomodulation in clinical and outpatient practices.

## 2 Method

### 2.1 Study design

**Figure 1:**
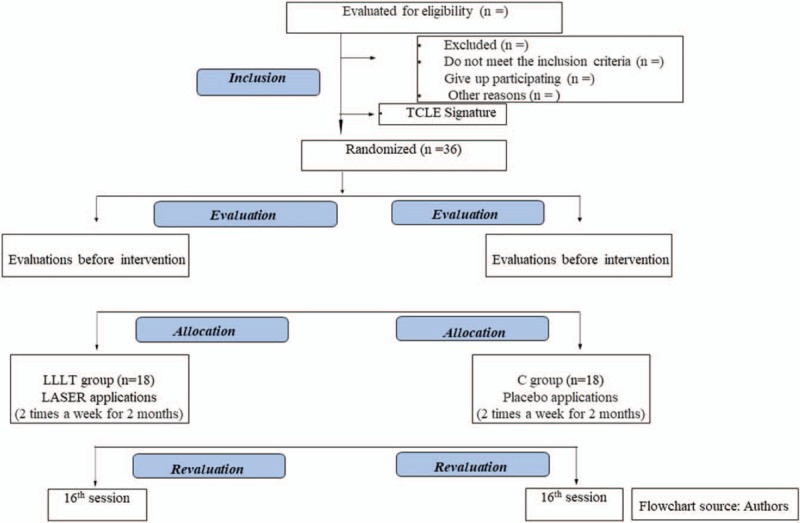
Flowchart of the study.

This will be a clinical, randomized, comparative, and blind study. There will be 36 patients randomized into 2 treatment groups: LLLT treatment or placebo (C) (Fig. 1). The study will be implemented over 2 months, divided into 16 sessions, 2 times a week. It will be conducted at the Medicine Outpatient Clinic of the Nove de Julho University, in the city of São Paulo, Brazil. This protocol was written and was based on the Standard Protocol Items: Recommendations for Interventional Trials (SPIRIT). The dissemination and the registration for participation in the study will be carried out through the Nove de Julho University website. The participants will be residents who will mainly be recruited from the city of São Paulo. The participants will be informed about the research, the procedures, the risks, and the benefits by FCFC (the author of this protocol). If they agree, they will sign the informed consent form (ICF). Only the participants, who after reading and agreeing to the protocol, and who sign the ICF, will be part of the study.

The study will start in March 2020. The study is not yet recruiting. After the recruitment, the researchers will check if the patients meet the inclusion/exclusion criteria based upon the anamnesis, the Berlin questionnaire, and the polysomnography evaluation.

The patients and/or the public will not be involved in the design, the recruitment, or the conduct of the study. The participants will be men and women who are sedentary, who are not on a diet or drug therapies, and who are at a risk of OSA, by screening with the Berlin questionnaire. Initially, they will perform nocturnal polysomnography. Those diagnosed with moderate and severe OSA (apnea/hypopnea index, AHI ≥15 events/h) will be invited to participate in the study.

### 2.2 Patient and public involvement statement

The patients and/or the public will not be involved in the design, the recruitment, or the conduct of the study. The participants will be men and women with moderate or severe obstructive sleep apnea who are sedentary, and who are not on diet or drug therapies.

### 2.3 Inclusion criteria

This study will be conducted on men and women aged 30 to 60 years, who are at a risk of OSA, by screening with the Berlin questionnaire. Those patients who have agreed to participate in the study will perform nocturnal polysomnography. Those diagnosed with moderate and severe OSA (apnea/hypopnea index, AHI ≥15 events/h) will be invited to participate in the study.

Subsequently, they will be submitted to clinical/physical examinations, with measurements of demographic and body composition (bioimpedance), as well as with blood pressure and blood tests. The blood samples will be collected from the venous blood after 12 hours of overnight fasting, in order to determine the total serum cholesterol, the triglycerides, the high-density lipoprotein (HDL) cholesterol (enzymatic method), and the plasma glucose (standard glucose oxidase method) concentrations.

### 2.4 Exclusion criteria

The participants will be excluded from the survey if they present any of the following:

- patients on a diet, on an exercise program, or on drug treatment;
- pediatric and elderly patients;
- patients with diagnosed central apnea;
- smokers;
- alcoholics;
- oncologists;
- pulmonologists;
- patients with heart disease;
- hypothyroidism;
- pregnant women;
- history of photosensitivity (allergies).

### 2.5 Sample size calculation

The sample size was calculated at http://www.openepi.com. It was determined according to an earlier study, with a similar population, displaying metabolic syndrome, and sleep apnea.^[6]^ The primary outcome was a reduction of the AHI. A power of 80% was considered, with a bicaudal type 1 error of 0.05, in order to detect a difference in the AHI of 16 events/h, assuming a standard deviation of 20 events/h.

### 2.6 Randomization

Through the website http://www.randomization.com/, a random block list was generated for the 2 studied groups, LLLT and C. The randomization plan was for 9 blocks of 4 individuals. For both of the groups, the order of the randomization will occur at the entrance to the protocol, by consulting the random list and indicating to which group the patient should be allocated.

### 2.7 Review descriptions

These will be the review descriptions: physical capacity (VO_2_ peak at the cardiopulmonary exercise test, CPET); hemodynamic responses during the CPET; vascular function (HR and PA, flow-mediated dilatation, FMD); autonomic (heart rate and blood pressure variability); sympathovagal balance; and baroreflex control at rest.

### 2.8 Interventions

**Figure 2:**
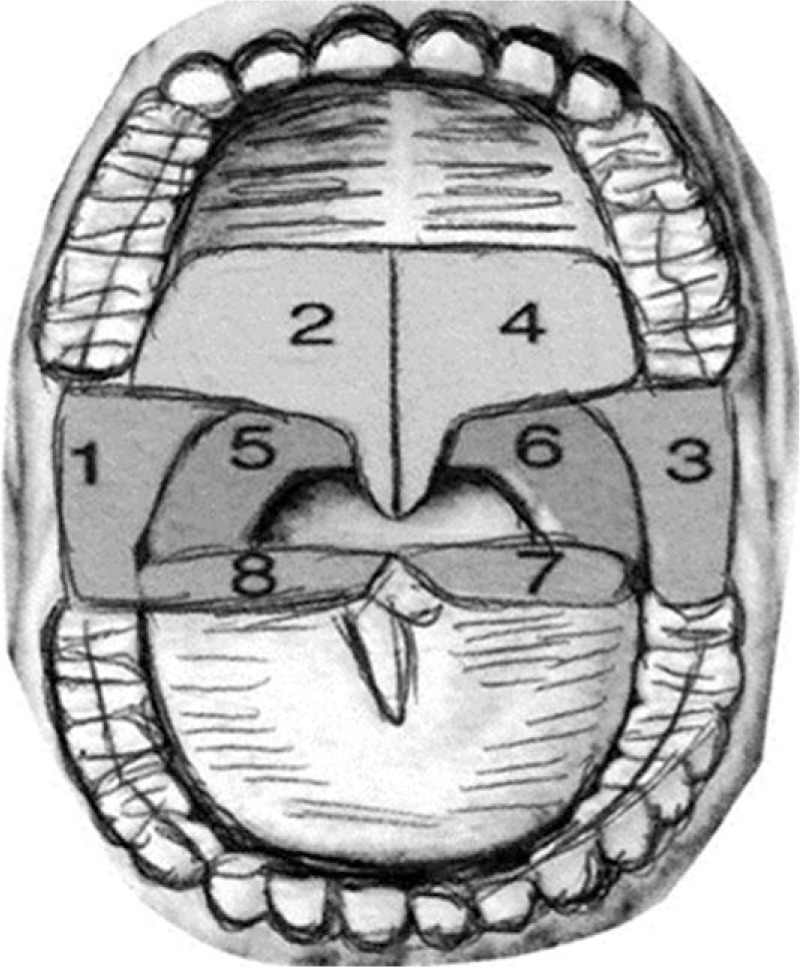
The 8 points of LLLT application: soft palate, uvula, pharyngeal walls, palatine tonsils, and on the tongue base. The LASER therapy will apply twice a week, over a 2-month period, totaling 16 sessions. Each point will be stimulated for 8 seconds in the soft palate, uvula, pharyngeal walls, palatine tonsils, and on the tongue base. Figure adapted from Storchi IF.^[4]^ LASER = light amplification by stimulated emission of radiation, LLLT = low-level laser therapy.

The LLLT group will receive the laser application at 8 points, twice a week, over a 2-month period, totaling 16 sessions. Each point will be stimulated for 8 seconds in the soft palate, uvula, pharyngeal walls, palatine tonsils, and on the tongue base (to the extent that the anatomy and compliance will allow). See Figure 2.

The laser used will be the Therapy Plus NS 13678 (Mark DMC, São Paulo, Brazil), with a spot diameter of 2.35 mm and a spot area of 0.0434 cm^2^. For that, the wavelength will be 808 nm (nanometers), with a power of 250 mW (milliwatts), and an energy of 2 J (joules). The LLLT application data is best specified in Table 1.

**Table 1:**
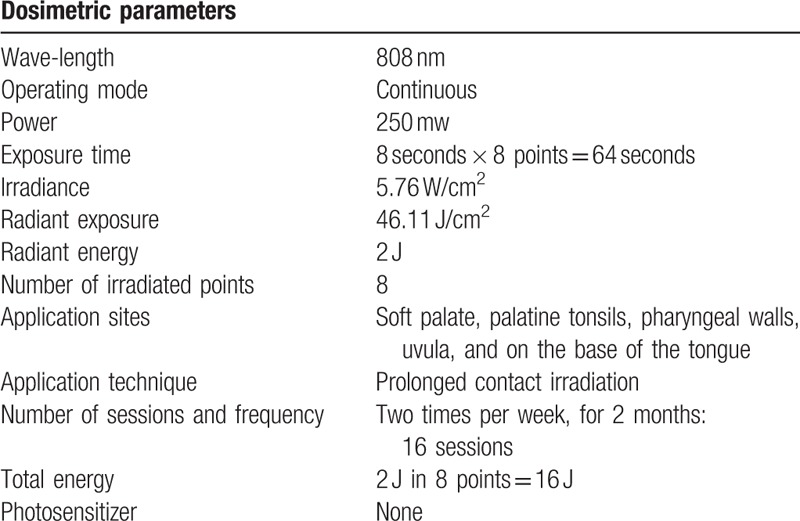
LLLT protocol applied by continuous infrared in patients with obstructive sleep apnea.

In the C group, they will receive placebo applications of the photobiomodulation, with only the use of a guiding light that is visually very similar to the LLLT, for the same 8 seconds and at the same 8 points, but without any effects, because the laser will be turned off.

During the ensuing routines, the patients will undergo 2 months of treatment and they will receive 16 sessions of photobiomodulation (2 times a week).

During the sessions, the researchers will remind the participants of the importance of adherence to the treatment sessions, in order to improve their compliance with the intervention protocol. The participants will be instructed to continue their usual routines for health conditions as usual.

### 2.9 Study variables

The primary variable of this study will be the AHI per hour of sleep. The secondary variables will be improvements in the autonomic and hemodynamic control of the subjects with OSA.

#### 2.9.1 Berlin questionnaire

The OSA screening will be conducted when using the Berlin Questionnaire (BQ). The patients with a high risk of OSA will be submitted to polysomnography. Before the evaluations of PSG, all of the patients will complete the 11-item self-reporting BQ (divided into 3 categories), with the aim of detecting important symptoms for the diagnosis of OSA. Those subjects that indicate in at least 2 positive categories will be considered at a high risk for OSA. In Category 1, a positive score will be defined as persistent symptoms (3–4 times/wk) in ≥2 questions about their snoring. In Category 2, a positive score will be defined as persistent (3–4 times/wk) for wake-time sleepiness, drowsy when driving, or both. In Category 3, a positive score will be defined as a history of high blood pressure or a body mass index (BMI) ≥30 kg/m^2^.^[7–9]^

#### 2.9.2 Polysomnography

There will be overnight polysomnography. The researchers will evaluate OSA by an overnight PSG (Night One Philips Respironics Digital System, 4 Channels, Philips Respironics, Philips Medical Devices) as previously described.^[5]^ The device is also equipped with software, in order to measure the relevant parameters of the PSG. The PSG will start at around 10:00 pm and will finish at around 6:00 am. The AHI will be calculated as the total number of respiratory events (apneas plus hypopneas), divided by the total sleep time, and expressed in events per hour (events/h). The AHI cutoff for OSA will be based on the Task Force of the American Academy of Sleep Medicine.^[5]^ The severity of OSA will be classified as such: non-OSA for AHI <5 events/h, mild OSA for AHI from 5 to 14.9 events/h, moderate OSA for AHI from 15 to 29.9 events/h, and severe OSA for AHI will be ≥30 events/h.^[5]^ Apnea will be defined as a 90% decrease of airflow for at least 10 seconds, while hypopnea will be defined by a >50% decline in airflow in respiratory signals for at least 10 seconds, accompanied by 3% oxygen desaturation.^[5]^ All of the equipment to be used is appropriate for the legal regulations of Brazil and the equipment will use standardized positioning, in order to minimize any experimental bias.

#### 2.9.3 Qualitative variables

- Anatomy of the oral cavity (Modified Mallampati Test)^[10]^
- Daytime sleepiness (Epworth Scale)^[11]^
- Quality of life (SF 36; Medical Outcome Study)^[12]^
- Stress level (the Brazilian version of the Perceived Stress Scale)^[13]^;
- Level of physical activity (International Physical Activity Questionnaire [IPAQ])^[14]^.

#### 2.9.4 Epworth sleepiness scale

The Epworth sleepiness scale (ESS) is a questionnaire to detect daytime sleepiness, consisting of a simple subjective scale, and covering 8 different daytime situations. The subjects will be asked to rate on a scale of 0 (those who would never doze) to 3 (high chances of dozing); how likely they would be to doze off or fall asleep in 8 situations, based on their recent everyday life. A distinction will be made between dozing off and simply feeling tired. Thus, the scale will be ranged from 0 to 24, and excessive daytime sleepiness will be defined when the score is ≥11.^[11,15]^

The questionnaire will take a full 10 minutes to be completed. The data that is to be collected for this study will only be administered by the principal investigators (the authors of this document).

### 2.10 Statistical analysis and data analysis plan

All of the personal records of the participants, such as the signed ICF, will be stored in locked file cabinets. The information will be transferred to a computer by FCFC (the author of this protocol) and the participants will be identified by ID numbers. All of this collected data will be stored on a university computer, protected by a password, and accessed by ICT (a co-author of this protocol) for the statistical analysis, or by the other researchers supervising this study. The Shapiro-Wilk test will be used to test the normality of the data. If the data are nonparametric, the normalization will be performed by a mathematical strategy. The Shapiro-Wilk test will be performed in order to analyze the normality of the distribution between the groups, and thus use the appropriate inferences tests. The descriptive data will be presented in relative frequency, with measures of a central tendency and dispersion, according to the nature of the variables, by a mean ± standard error, or by a median and interquartile interval.

Assuming the normality of the data, the characteristics of the groups at the pre-intervention time will be compared by the Student *t* test for the independent samples. For the analysis of the effects of the laser applications, a comparison of the means through the analysis of variance (ANOVA) for repeated measurements will be performed, associated with the post-hoc test when the effect is verified. Where necessary, nonparametric equivalent tests shall be used. The ANOVA test for the independent variables, followed by Bonferroni post-hoc test, will be used for the inferential analysis. A *P* < .05 value will be considered statistically significant.

The analyses will be performed in the Statistic Package for the Social Science—IBM SPSS Statistics for Windows, Version 20.0 (IBM Corp, Armonk, NY). for all of the calculations, being adopted to a level of significance of 5%.

## 3 Expected results

The researchers hope to identify whether there will be a decrease in the AHI by the polysomnography measurements when applying an 808 nm infrared laser photobiomodulation by the Therapy Plus equipment.

These measurements will be performed prior to, and after, the 16 treatment sessions. At the primary endpoints and at the secondary endpoints, the researchers expect to verify a decrease in apnea, as well as in the hypopnea index and snoring, with improvements in the autonomic and hemodynamic control of the subjects with OSA. Finally, the study expects to verify improvements in sleep and in the patients’ quality of life.

## 4 Discussion

This current work is presently describing a study protocol for a randomized, blind, clinical trial, in order to study and evaluate the AIH, based upon a comparison between an LLLT intervention and a placebo intervention, from 2 different groups. These being: a photobiomodulation group; a sham photobiomodulation group. The results of this study are expected to provide evidence on the role of phototherapy in conditions of OSA.

In this protocol, the choice of the photobiomodulation parameter was made from studies that have demonstrated that an application of laser on the soft palate of patients, a region more susceptible to collapse during sleep, could trigger a proliferation of cells. This would be with an increase of the collagen fibers, as well as with an increase in the circulation, modulating the processes of the inflammatory conditions. In fact, an experimental study found decreased inflammation when using low-intensity laser therapy.^[2]^

The patients who have a difficulty to adapt may benefit from a new therapy. In fact, although CPAP has proven its effectiveness, it is a palliative treatment. In this sense, LLLT, if it proves to be efficient, could be an adjuvant therapy to the conventional treatments with CPAP and the intra-oral device. LLLT may be a new therapeutic option that is safe, easy to apply, and inexpensive (when compared with CPAP and even intra-oral). In addition, it has been demonstrated in mice that LLLT in the vascularized region increases the parasympathetic activity, generating a deceleration of the heart rate, a decrease in blood pressure, a decrease in the sympathetic nervous activity, and a decrease in blood glucose. There is a strong association of OSA with snoring, obesity, and systemic arterial hypertension (SAH), predisposing these patients to a greater cardiovascular risk.

The researchers will test the hypothesis that LLLT, when applied to the soft palate, decreases the phallus collapsibility, snoring, and the apnea/hypopnea index. In addition, to see whether it improves the autonomic and hemodynamic control in patients with moderate and severe OSA.

Recently, a new and preliminary Erbium YAG laser treatment has been shown to be effective in the reduction of snoring and it has achieved a satisfaction rate of 65%. This is in addition to discreetly improving the rate of apnea and hypoxia, sleep quality, and respiration, when applied on the soft palate, uvula, palatine tonsils, and on the base of the tongue. The mechanisms of action of the Erbium YAG LASER generate a shrinkage of the collagen fibers in the oral mucosa, and they trigger a neo-collagenase action.^[4]^

The infrared low-intensity laser (808 nm) is absorbed by the substances that are present in the plasma membranes of the deeper cells, such as in the connective, muscular, bone, and cartilaginous tissues. It alters the membrane permeability with an increased absorption of nutrients, water, and dermo-cosmetics. It activates the cellular metabolism and it increases the microcirculation while stimulating the collagen fibers. It also has anti-inflammatory, analgesic, cicatrizing effects, and it improves autonomic control.^[3]^

Based on these considerations, in this work, the researchers wish to observe if there is an increase in the percentages of satisfaction in the patients with OSA, by using a different approach to the treatment, based on the patient's selection, when using validated questionnaires, polysomnography, and spirometry. Thus, the study will investigate whether low-intensity laser photobiomodulation when applied to patients who snore and with OSA, produces local, autonomic, and blood inflammatory markers, respectively, through evaluations and re-evaluations of the sleep study, the upper airway collapsibility, the parameters that reflect hemodynamics, and the blood markers.

The inclusion and the exclusion criteria for this study have been based on the literature that has objectively formulated what needs to be chosen as the basis for a good study, such as the use of medications, or the patient's ease of following the necessary procedures, in order to achieve an effective result of the treatment applied.

The strength of this study will be the presence of the intervention group and the control group since the interventions will be evaluated at the beginning, and at the end of the study.

The limitations of this study may be related to the lifestyle habits and the routines of the participants. These procedures can affect the results, as a consequence of their daily stress, their food, their cosmetics use, and their photo exposures.

To the best of the researchers’ knowledge, no previous randomized control trial has evaluated whether this type of phototherapy is effective. The currently proposed trial is the first randomized clinical trial study to evaluate the role of phototherapy in a sleep apnea condition. In fact, the results of this study will provide valuable clinical evidence for an objective assessment of the potential benefits and the risks of the stated procedures. The final results will be published at the end of the study.

## 5 Other information—ethics and disclosure

This protocol was approved by the Research Ethics Committee of the Nove de Julho University, São Paulo, Brazil, dated March 11, 2019 (CAAE: 06025618.2.0000.5511)—Acceptance Number: (3.191.077), including the Informed Consent Form. The trial has already been registered at the Brazilian Registry of Clinical Trials (REBEC - RBR-42v548), and it was first registered on June 04, 2019, UTN: U1111-1230-9271, which provides public access to the full protocol. All of the items from the World Health Organization Trial Registration Data are available on the REBEC (Registro Brasileiro de Ensaios Clínicos) register website. Any modification regarding the study's protocol will be sent to the Research Ethics Committee of the Nove de Julho University as an amendment, and an updated version will be sent to REBEC. After the publication of the protocol, the data will be collected and the results will be presented at meetings, and then published in a scientific journal, selected by an area of interest and upon the impact factor. At the end of the study, the main results will be shared with the participants by email. The authorship of the results manuscript will include the authors of the protocol, together with others who may contribute to the procedures or to the data analysis.

## Acknowledgments

The authors would like to thank the group of teachers and researchers from the Universidade Nove de Julho, UNINOVE, Brazil. Without the support of these teachers and researchers, inevitably, the formulation of this protocol would not have even been possible.

## Author contributions

**Conceptualization:** Fernanda Cristina Ferreira-Camargo, José Roberto De Moura, Felipe Xerez Cepeda, Marilia de Almeida Correia, Ivani Credidio Trombetta.

**Data curation:** Fernanda Cristina Ferreira-Camargo, José Roberto De Moura, Marilia de Almeida Correia, Ivani Credidio Trombetta.

**Formal analysis:** Fernanda Cristina Ferreira-Camargo, Ivani Credidio Trombetta.

**Funding acquisition:** Fernanda Cristina Ferreira-Camargo, Ivani Credidio Trombetta.

**Investigation:** Fernanda Cristina Ferreira-Camargo, José Roberto De Moura, Marilia de Almeida Correia, Ivani Credidio Trombetta.

**Methodology:** Fernanda Cristina Ferreira-Camargo, Ivani Credidio Trombetta.

**Project administration:** Fernanda Cristina Ferreira-Camargo, Ivani Credidio Trombetta.

**Resources:** Fernanda Cristina Ferreira-Camargo, Marilia de Almeida Correia, Ivani Credidio Trombetta.

**Software:** Fernanda Cristina Ferreira-Camargo, Ivani Credidio Trombetta.

**Supervision:** Fernanda Cristina Ferreira-Camargo, José Roberto De Moura, Felipe Xerez Cepeda, Marilia de Almeida Correia, Ivani Credidio Trombetta.

**Validation:** Fernanda Cristina Ferreira-Camargo, José Roberto De Moura, Marilia de Almeida Correia, Ivani Credidio Trombetta.

**Visualization:** Fernanda Cristina Ferreira-Camargo, José Roberto De Moura, Marilia de Almeida Correia, Ivani Credidio Trombetta.

**Writing – original draft:** Fernanda Cristina Ferreira-Camargo, Marilia de Almeida Correia, Ivani Credidio Trombetta.

**Writing – review & editing:** Fernanda Cristina Ferreira-Camargo, Ivani Credidio Trombetta.
